# Radial Extracorporeal Shockwave Therapy versus Ultrasound Therapy in Adult Patients with Idiopathic Scoliosis

**DOI:** 10.3390/jcm10081701

**Published:** 2021-04-15

**Authors:** Cristina Daia, Cristian Scheau, Corneliu Toader, Ana Maria Bumbea, Visarion Danut Caimac, Ioana Andone, Cristina Popescu, Aura Spanu, Gelu Onose

**Affiliations:** 1 Department of Medical Rehabilitation, “Carol Davila” University of Medicine and Pharmacy, 041914 Bucharest, Romania; cristinaoctavianadaia@gmail.com (C.D.); corneliutoader@gmail.com (C.T.); ioanaandone11@yahoo.com (I.A.); cristina_popescu_recuperare@yahoo.com (C.P.); aura_ko@yahoo.com (A.S.); geluonose@gmail.com (G.O.); 2 Neuromuscular Department, Clinical Emergency Hospital “Bagdasar Arseni”, 041914 Bucharest, Romania; 3 Department of Physiology, “Carol Davila” University of Medicine and Pharmacy, 050474 Bucharest, Romania; 4 Department of Neurosurgery, National Institute of Cerebro-Vascular Diseases, 041914 Bucharest, Romania; 5 Department of Medical Rehabilitation, University of Medicine and Pharmacy, 200349 Craiova, Romania; anamariabumbea@yahoo.com (A.M.B.); dcaimac@yahoo.com (V.D.C.); 6 Neurorehabiltation Department, Clinical Neuropsychiatry Hospital, 200473 Craiova, Romania

**Keywords:** radial extracorporeal shockwave therapy, ultrasound therapy, adult idiopathic scoliosis, pain, quality of life, disability

## Abstract

Background: This study aimed to compare the effectiveness of radial extracorporeal shockwave and ultrasound therapies in adult patients with idiopathic scoliosis in terms of pain, disability, and quality of life. Methods: Forty-eight patients with idiopathic scoliosis were randomly divided into three groups of 16: shockwave, ultrasound, and control. The patients were evaluated at admission (day one) and at discharge (day 14) for pain, by using the visual analogue scale; for disability, by using the Oswestry disability index; and for the quality of life, with short form-36. Results: Radial extracorporeal shockwave therapy was more effective than ultrasound in reducing pain (*p* = 0.004) and increasing quality of life, bringing extra vitality (*p* = 0.003) and emotional comfort (*p* = 0.007) to the patient. Both shockwave therapy (*p* = 0.001) and ultrasound therapy (*p* = 0.003) were effective in reducing pain. In terms of disability, both treatments had similar effects (*p* = 0.439). Conclusion: Radial shockwave was significantly more effective than ultrasound in reducing pain and increasing the quality of life, bringing additional vitality and emotional comfort to the patient with idiopathic scoliosis. In terms of disability, both treatments had similar effects when associated with kinesitherapy.

## 1. Introduction

Idiopathic scoliosis is a three-dimensional spinal deviation of the spinal axis with a curvature exceeding 10° on a plain anteroposterior X-ray image, with no other underlying disease identified to provoke it [1]. It is the most common spinal deformity that develops in otherwise healthy children [2]. A proportion of 23% to 51% of adolescents with idiopathic scoliosis had significant back pain [2,3]. Back pain is approximately twice as prevalent in adolescents with idiopathic scoliosis compared with non-scoliosis patients [4], and it decreases the patients’ quality of life [5]. Literature data regarding pain in adults with idiopathic scoliosis are scarce; however, a main cause of pain in idiopathic scoliosis is muscle contracture, mainly located in the convexity of the spine [6]. Adults with idiopathic scoliosis have back pain generated by self-sustaining muscle contractures [7] induced by movements; hence, it is important to have an effective and secure therapeutic approach with muscle-relaxing effects such as physiotherapy procedures, as in as shockwave (SW) [7] or ultrasound (US) therapies [8], which are two related procedures in terms of mechanical and physical properties.

Oscillatory electro-mechanic therapy is the application of oscillation waves with high frequency and pressure, which relies on a mechanical means of propagation. In theory, this category includes ultrasonic waves and ballistic shock waves. Ultrasound is a mechanical oscillation produced by physiotherapy devices based on the inverted piezoelectric effect, which spreads within tissues with high frequencies in the range of 8 × 10^5^–1 × 10^6^ Hz [9,10,11]. Therapeutic dosing of US is carried out according to power density (W/cm^2^), application time (minutes), and pulse application form (continuous/burst) [9,10,11].

Shockwaves are a complex of “high-pressure” oscillation waves that yield around 150 mega Pascals (MPa), causing real mechanical shocks in the targeted tissues. Alternate names such as “high-pressure waves”, “ballistic waves”, or “shockwaves” were assigned for this very reason, as they cause micro-trauma [12,13]. There are multiple means of producing SWs. One method is based on the electrohydraulic effect, resulting in low-pressure SWs, also known as radial shockwaves (RSW). Another manner of generating SWs relies on the electromagnetic effect, the inverted piezoelectric effect, and a spark discharge, producing high-pressure SWs, labeled focused shockwaves. Shockwave therapy dosing factors in the number of shocks, application time, frequency, energy transfer, and form of application (continuous or burst) [12].

Both US and SWs produce a mechanical impact on the target tissue, causing similar physical and chemical effects, such as heat generation, cavitation, and diffusion. The two types of waves also have common clinical effects, mainly, pain relief and muscle relaxation [14,15]. However, the pressure level at which these effects occur is different, as SWs have a pressure peak of at least 1000 times stronger than that of US [16,17]. Therefore, patient pathology plays an important role in choosing the appropriate method [18,19].

The effectiveness of US and SWs has been compared in pathologies such as fibromyalgia/myofascial pain syndrome [20], plantar fasciitis [21,22], epicondylitis [23,24], and tendonitis of the shoulder [25], and no significant differences were noted in terms of alleviating pain, increasing the quality of life (using the short form-36 (SF-36)), or improving disability. In our paper, we aim to measure the effectiveness of these two treatment modalities in terms of improving the pain, disability, and quality of life in adult patients with idiopathic scoliosis.

## 2. Materials and Methods

The study comprises 48 patients diagnosed with idiopathic dorsal-lumbar scoliosis, either admitted to the hospital or benefiting from ambulatory care, between February 2019 and February 2020.

### 2.1. Inclusion Criteria

- Patients over 30 years old;
- Low back dorsal-lumbar nonspecific musculoskeletal pain;
- Diagnosed with dorsal-lumbar idiopathic scoliosis of any type (mild, moderate, or severe) and any degree of curvature from 10 to 60 degrees.

### 2.2. Exclusion Criteria

Our study employed the classical contraindications for any electrotherapy procedure [7]:

- Idiopathic scoliosis that required surgery intervention;
- Any vertebral or spinal bone marrow pathology other than idiopathic scoliosis: disc herniation, spinal cord injury, benign or malignant tumors, and so on;
- Fever, regardless of the cause;
- Acute or decompensated diseases, regardless of their nature: infectious, inflammatory, cardiovascular, respiratory, neurological, rheumatological, metabolic, and so on;
- Uncontrolled hypertension with systolic blood pressure higher than 150 mmHg;
- Any mental disorder;
- Any neoplasm;
- Osteosynthesis materials or other metals in the area of application;
- Patients carrying a cardiac pacemaker;
- Pregnant women, women during menstruation, women with intrauterine devices (IUD);
- Patients with altered general condition or cachexia;
- Patients with any pathology that could generate a disability other than idiopathic scoliosis;
- Patients with any type of idiopathic scoliosis other than dorsal-lumbar;
- Patients with any pain other than low-back dorsal-lumbar pain.

### 2.3. Study Design

In our study, we used block randomization to obtain three balanced groups. We set block randomization for gender (male and female), scoliosis type (dorsal and lumbar; then, left and right curvatures), curves in degree categories (0–10, 11–20, 21–30, 31–40, 41–50, 51–60), and age groups (30–40, 41–50, 51–60, 61–70, 71–80) (Table 1). We balanced the number of subjects in each block in order to increase the comparability between the groups.

Group A (*n* = 16) received US treatment (UST), applied bilaterally in the paravertebral dorsal-lumbar region. The regimen was 0.5 W/cm^2^, for 5 min, using continuous emission, dynamic application in a slow rhythm, and daily application rhythm over a total of 10 sessions. Group B (*n* = 16) received RSW treatment (RSWT) to the paravertebral dorsal-lumbar area using a pressure of 0.2 MPa (2 Barr) in addition to a series of 2000 shocks in pulse emission (BURST mode) with a frequency of 10 Hz and an average energy transfer of 0.4 m J/mm^2^ for a duration of 4.48 min. The application rate was every two days with a total of five sessions. Radial extracorporeal shockwave therapy was delivered every two days in order to avoid microtrauma cumulating effects. Group C (*n* = 16) was the control group and did not receive physiotherapy. The total treatment time was 14 days.

### 2.4. Patient Rehabilitation Management

Each patient signed a detailed informed consent. The study respects the ethical principles for research presented in the Declaration of Helsinki. All patients included in this study underwent adequate treatment in compliance with ethical guidelines, standing good practices, and associated comorbidities. All patients benefited from the same kinesitherapy procedures as part of the rehabilitation program. The kinesitherapy program was provided by a kinetotherapist and consisted of a sum of specific exercises named the Klapp method [27]. All patients benefited from the same exercise program, applied daily for the same period of 10 days, 60 min per day.

### 2.5. Patient Evaluation

All patients were evaluated according to the national protocol of evaluation in non-traumatic spine pathologies [28]. The clinical and paraclinical evaluation consists of recording and describing six specific syndromes through various diagnostic methods: (1) spinal, active, and passive (posture and radiography); (2) muscle (contracture, ultrasonography [29]); (3) radicular (neurologic examination and MRI); (4) spinal cord (neurologic examination); (5) psychological (psychological examination); and (6) functional (disability evaluation) [28]. The pain experienced by the patients in our study was caused by muscle contraction, and this was a criterion for enrollment in this study. The kinesitherapy program was administrated concomitant with the interventions, for all groups.

### 2.6. Evaluated Parameters

For all groups, the total time for evaluation was 14 days. The following parameters were assessed:

1. Pain: using the visual analogue scale (VAS), which highlights 10 degrees of pain from 0 (no pain) to 10 points (the most severe pain) [30]. The VAS scale was performed on days 1 and 14 for all patients, before and after the application of US and RSW for the study groups.
2. Disability: using the Oswestry disability index (ODI). The ODI scale was performed on days 1 and 14 for all patients [31].
3. Quality of life (QoL): using short form-36 (SF-36), which consists of 8 subscales with 36 items that evaluate the physical, social, and usual activity limitations due to physical or emotional problems, bodily pain, mental health, vitality, and general health perceptions [32]. The QoL survey was performed only on day 14 for all patients.

### 2.7. Statistical Analysis

SPSS Statistics for Windows version 15.0 (SPSS Inc., Chicago, IL, USA) was used for statistical analysis. The distribution of patient demographic features was analyzed using descriptive statistical methods. Population normality was assessed using the Kolmogorov–Smirnov test. Levene’s test for equality of variances, *t*-test for equality of means, the Mann–Whitney test, and the Wilcoxon test were used for comparison of parameters. The Tamhane post hoc evaluation was employed after multiple comparisons were performed by ANOVA when equal variances were not assumed. Pearson’s chi-squared test was used for comparing categorical data. The statistical significance of *p*-value was considered *p* < 0.05. For the Kolmogorov–Smirnov test, *p*-value was considered *p* > 0.2.

## 3. Results

The demographic analysis of the study groups showed that the patients were similar in terms of age (F = 0.07, sig. 0.926, ANOVA) and gender (0.182 Pearson chi-square, sig. 0.913) distribution (Table 2).

The analysis of the initial VAS in all lots shows similar values (0.485, ANOVA; 0.963, Tamhane post hoc). The evaluation of pain on the VAS scale shows that the average differences in the final VAS (compared with the initial score) were significantly lower in the RSWT lot (−6.38 ± 1.02) compared with the UST lot (−5.38 ± 0.71) and the control group (−4.50 ± 0.62), with a statistical significance of *p* < 0.001 and *p* = 0.004, respectively (Mann–Whitney). The difference in the VAS score was also significantly lower in the UST patients compared with the control patients (Table 3).

The analysis of the initial ODI in all lots shows a comparable value (0.465 ANOVA, and 0.981 Tamhane post hoc). Regarding the evaluation of disability using ODI (highlights), the average of the differences between the final and the initial ODI in the RSWT group (−28.06 ± 4.32) did not differ significantly from that of the UST group (−29.56 ± 6.29) or the control group (−27.31 ± 4.743) (*p* = 0.361 Mann–Whitney, *p* = 0.439, ANOVA, post hoc Tamhane 0.955).

Regarding the QoL, the SF-36 subscales showed significantly better values for the following parameters: vitality (means 21.22 versus 11.78; *p* = 0.003) and emotional component (means 20.84 versus 12.16; *p* = 0.007) in the RSWT group versus UST; no other statistically significant differences were noted (Table 4).

No significant immediate or delayed adverse reactions were observed following the physiotherapy procedures.

## 4. Discussion

In this study, we used five large block randomizations to increase the comparability between groups by keeping the ratio of the number of subjects between them almost the same. Consequently, the risk of selection bias was increased as the treatment of the subject in the block was known. Because we analyzed a new physiotherapy treatment, we chose this type of randomization in order to recognize its therapeutic benefits, starting from similar lots, to the best of our ability [33]. Although randomization minimizes the selection bias in this case–control study, it was not a blinded intervention; therefore, another limitation of this study was allowing the subsequent differential cointerventions or biased assessment of outcomes [34]. The number of cases in the analyzed groups is low; therefore, the results we share through this study should be considered as indicative while encouraging subsequent research to verify them on larger populations. For the symptomatic treatment of idiopathic scoliosis, we chose to compare two methods of physiotherapy with similar features, as they both rely on mechanical waves. Participants had low-back muscle pain caused by prolonged muscle contracture, especially from the convexity of scoliosis. Patients had no headache, sore areas, spinal pain, generalized pain, or of any other type. The selection of a clear type of pain (muscle contraction pain), due to a specific type of idiopathic scoliosis (dorsal-lumbar scoliosis), could be another limitation of the antalgic potential of the proposed physiotherapy: RSWT and UST. On the other hand, we demonstrated that RSWT and UST have significant statistical antalgic effects acting on muscle contractures; therefore, we can recommend this kind of physiotherapy for this specific kind of pain. The main limitation of this study should be considered the lack of a standard protocol regarding RSWT and UST in scoliosis. To our knowledge, this is one of the first studies in the field, and the results appear promising. However, more studies on a larger number of patients are needed to confirm these results. Another limitation is the short-term evaluation of the outcomes assessed in the study. In our view, a follow-up examination would be the next step to be addressed in further studies.

Analyzing the results, we observed that RSWT produces a statistically significant decrease in pain generated by idiopathic scoliosis versus UST. It should be noted, however, that both treatments determined an average decrease in the VAS score for pain: UST by 5.38 points, and RSWT by 6.38 points, respectively; however, RSWT proved to be statistically more effective and was further followed by a significantly higher state of emotional comfort and vitality according to the analysis of the patients’ QoL. Regarding the rest of the parameters analyzed in SF-36, we did not detect any other statistically significant differences. The mental health component of QoL SF-36 was not analyzed because the presence of mental illness was considered an exclusion criterion in our study; moreover, according to literature data, mental health factors do not influence QoL SF-36 in patients with scoliosis [35]. Radial extracorporeal shockwave therapy has been demonstrated to have a significant influence on the reduction of pain in patients with chronic low-back pain [36]. The statistically proven superior analgesic effect of RSWT may be explained by its particular mechanism of destroying unmyelinated sensory fibers [37] added to fibrinolytic properties [38] when compared to UST [39], determining the lysis of fibrin bridges in long-lasting scoliosis contractures, while also enabling effective muscle relaxation and subsequent participation in movement.

Regarding the disability induced by idiopathic scoliosis in adults [40], which was investigated in our study using ODI, there were no statistically significant differences in its improvement after therapy. This finding is consistent with the fact that all patients benefited from a similar kinetic program, an element with an essential role in terms of patient functionality [41].

Both types of physiotherapy were very well-tolerated by patients, who showed no side effects; therefore, we recommend both methods in terms of safety and tolerability profile.

## 5. Conclusions

After a short-term evaluation, radial extracorporeal shockwave therapy and ultrasound therapy were both effective in reducing pain due to muscle contraction in adult patients with idiopathic scoliosis; however, RSWT had statistically superior effects in reducing pain and increasing the quality of life, as well as bringing additional vitality and emotional comfort to the patients. In terms of disability, both treatments have similar effects when associated with kinesitherapy. Both types of physiotherapy are safe and well-tolerated by patients with idiopathic scoliosis. Further studies involving larger study groups and follow-up examinations are needed in order to extend and confirm our reported findings, as well as to develop appropriate standardized protocols for these types of patients.

## Figures and Tables

**Table 1:** Patient characteristics in the three study lots regarding scoliosis type, degree, and severity.

RSWT ^1^ Lot				UST ^2^ Lot				Control Lot			
**Patient No.**	Dorsal Lumbar Scoliosis Type	Degree Curve	Severity Type ^3^	Patient No.	Dorsal Lumbar Scoliosis Type	Degree Curve	Severity Type ^3^	Patient No.	Dorsal Lumbar Scoliosis Type	Degree Curve	Severity Type ^3^
1	left	35	moderate	17	left	32	moderate	33	left	50	severe
2	right	55	severe	18	left	20	mild	34	left	30	moderate
3	left	38	moderate	19	right	38	moderate	35	left	20	mild
4	right	38	moderate	20	right	32	moderate	36	right	35	moderate
5	right	38	moderate	21	right	55	severe	37	right	55	severe
6	left	50	severe	22	right	35	moderate	38	right	50	severe
7	right	35	moderate	23	right	55	severe	39	right	22	mild
8	right	18	mild	24	left	30	moderate	40	right	35	moderate
9	left	20	mild	25	left	48	severe	41	left	45	severe
10	left	50	severe	26	left	50	severe	42	left	50	severe
11	left	45	severe	27	right	22	mild	43	right	38	moderate
12	left	30	moderate	28	right	38	moderate	44	right	30	moderate
13	right	50	severe	29	left	36	moderate	45	left	38	moderate
14	right	35	moderate	30	left	50	severe	46	left	32	moderate
15	right	55	severe	31	right	30	moderate	47	right	38	moderate
16	right	30	moderate	32	right	50	severe	48	right	50	severe

^1^ RSWT = radial extracorporeal shockwave treatment. ^2^ UST = ultrasound treatment. ^3^ Severity type: mild, 10–25; moderate, 26–40; and severe, over 40 [26].

**Table 2:** Demographic data of age and gender.

Lot	Demographic Data of Age						Demographic Data of Gender	
	*n*	Median	Mean	Min	Max	Std. Dev	Female Number	Male Number
RSWT ^1^	16	46.50	51	31	78	14.43	10	6
UST ^2^	16	51.00	51	32	74	12.18	11	5
Control	16	47.00	50	36	74	12.21	10	6
Total	48	50.85	51	31	78	12.72	31	17

^1^ RSWT = radial extracorporeal shockwave treatment. ^2^ UST = ultrasound treatment.

**Table 3:** Pain and disability index reported using the VAS and ODI questionnaire.

Test Parameters		VAS ^1^ Evolution			ODI ^2^ Evolution		
		RSWT Lot	UST Lot	Control Lot	RSWT ^3^ Lot	UST ^4^ Lot	Control Lot
No.		16	16	16	16	16	16
Normal Parameters	Mean	−6.38	−5.38		−28.06	−29.56	−27.31
	Std. Deviation	1.025	0.719		4.328	6.293	4.743
Most Extreme Differences	Absolute	0.232	0.308				
	Positive	0.232	0.308				
	Negative	−0.205	−0.199				
Kolmogorov–Smirnov Z		0.929	1.231				
Asymp. Sig. (2-tailed)		0.354	0.097				
Mann–Whitney U test mean rank		9.53	11.69	21.31			
Asymp. Sig. (2-tailed)		0.000	0.002				
Exact Sig. (2×(1-tailed Sig.))		0.000	0.003				
ANOVA Sig.					0.465		
Tamhane Sig.					0.823	0.823	0.955

^1^ VAS = visual analogue scale. ^2^ ODI = Oswestry disability index. ^3^ RSWT = radial shockwaves treatment. ^4^ UST = ultrasound treatment.

**Table 4:** Quality of life, short form-36, subscales RSWT versus UST lots—Mann–Whitney and Wilcoxon tests.

Statistic Test	General Health	Physical Functioning	Bodily Pain	Physical Role	Vitality	Role Emotional	Social Functioning
Mann–Whitney U	111.500	85.500	109.500	98.000	52.500	58.500	76.000
Wilcoxon W	247.500	221.500	245.500	234.000	188.500	194.500	212.000
Z	−0.626	−1.624	−0.710	−1.154	−2.865	−2.648	−2.000
Asymp. Sig. (2-tailed)	0.531	0.104	0.478	0.249	0.004	0.008	0.046
Exact Sig. [2 × (1-tailed Sig.)]	0.539	0.110 ^a^	0.491	0.270	0.003	0.007	0.051

^a^ Not corrected for ties. Grouping variable: TTip.

## Data Availability

The data sets used and analyzed during the current study are available from the corresponding author upon reasonable request. The data are not publicly available due to privacy reasons.
